# Unsupervised clustering of wildlife necropsy data for syndromic surveillance

**DOI:** 10.1186/1746-6148-6-56

**Published:** 2010-12-16

**Authors:** Eva Warns-Petit, Eric Morignat, Marc Artois, Didier Calavas

**Affiliations:** 1 Laboratory Environment and Prediction of Population Health, VetAgro Sup, Veterinary campus of Lyon, 1 avenue Bourgelat, BP 83, F-69280 Marcy-l'Etoile, France; 2 Epidemiology Unit, French Agency for Food, Environmental and Occupational Safety, 31 avenue Tony Garnier, F-69364 Lyon Cedex 07, France

## Abstract

**Background:**

The importance of wildlife disease surveillance is increasing, because wild animals are playing a growing role as sources of emerging infectious disease events in humans. Syndromic surveillance methods have been developed as a complement to traditional health data analyses, to allow the early detection of unusual health events. Early detection of these events in wildlife could help to protect the health of domestic animals or humans. This paper aims to define syndromes that could be used for the syndromic surveillance of wildlife health data. Wildlife disease monitoring in France, from 1986 onward, has allowed numerous diagnostic data to be collected from wild animals found dead. The authors wanted to identify distinct pathological profiles from these historical data by a global analysis of the registered necropsy descriptions, and discuss how these profiles can be used to define syndromes. In view of the multiplicity and heterogeneity of the available information, the authors suggest constructing syndromic classes by a multivariate statistical analysis and classification procedure grouping cases that share similar pathological characteristics.

**Results:**

A three-step procedure was applied: first, a multiple correspondence analysis was performed on necropsy data to reduce them to their principal components. Then hierarchical ascendant clustering was used to partition the data. Finally the k-means algorithm was applied to strengthen the partitioning. Nine clusters were identified: three were species- and disease-specific, three were suggestive of specific pathological conditions but not species-specific, two covered a broader pathological condition and one was miscellaneous. The clusters reflected the most distinct and most frequent disease entities on which the surveillance network focused. They could be used to define distinct syndromes characterised by specific post-mortem findings.

**Conclusions:**

The chosen statistical clustering method was found to be a useful tool to retrospectively group cases from our database into distinct and meaningful pathological entities. Syndrome definition from post-mortem findings is potentially useful for early outbreak detection because it uses the earliest available information on disease in wildlife. Furthermore, the proposed typology allows each case to be attributed to a syndrome, thus enabling the exhaustive surveillance of health events through time series analyses.

## Background

The importance of monitoring wildlife health is increasingly recognised [1,2], because free-ranging wild animals are victims, reservoirs or indicators of an increasing number of disease agents shared with humans and/or domestic animals [3-7].

General wildlife disease surveillance is a means of maintaining vigilance against emerging wildlife-related diseases [8,9], but it produces data that are frequently biased [10]. These data are further characterised by the diversity of monitored parameters: species, pathogens, diagnoses, environmental characteristics, etc. The analysis of data from this type of surveillance is usually limited to retrospective descriptive assessments. Passively acquired wildlife accessions may however also give insight into the occurrence of disease processes, whose significance may only become apparent over time [8]. Therefore, there is a need to monitor wildlife diseases prospectively, using an approach that takes into account the great diversity of the parameters.

Syndromic surveillance "applies to surveillance using health-related data that precede diagnosis and signal a sufficient probability of a case or an outbreak to warrant further public health response" (Center for Disease Prevention and Control, http://www.cdc.gov/ncphi/disss/nndss/syndromic.htm[11]). It has been developed in recent years in human health surveillance systems as a means of timely detection of disease outbreaks using robust pre-diagnostic data, which are registered automatically [12,13]. For efficient syndromic surveillance, it is necessary to group cases that share the same health indicators, in order to enhance the efficiency of event detection [14]. Health problems for which syndromic surveillance is used are either classified by bodily system [9,12,15,16] or focus on specific diseases, such as "influenza-like-illness" [17,18]. Syndrome definitions (groups of health indicators linked to these classifications) are either based on expert knowledge or on statistical classifications [12,13,19-21].

Macroscopic *post-mortem *findings are the primary data collected from cases of general wildlife disease surveillance. These descriptions form robust and reliable information, provided examinations are performed by experienced staff [22].They are also the only information available for diseases of unknown aetiology [8]. Diagnoses of causes of death are generally not available soon enough to assist early detection, because they depend on laboratory analyses that are costly, time consuming or unavailable [23,24]. Descriptions of macroscopic lesions can be used for the syndromic classification of cases [15]. Syndromic groups can be monitored over space and time for trend analysis and rapid detection of unusual health events, and can enhance the usual data analysis and its usefulness [8].

A general wildlife mortality monitoring network in France [25] has been compiling health data for over 20 years, including descriptions of necropsy findings. We chose to adapt the principles and methods of syndromic surveillance to these wildlife surveillance data. Syndrome definition is the scope of this paper, and our aim was to retrospectively identify and characterise distinct pathological profiles from these data which could be used to structure the whole dataset and thus take every case into consideration. Clustering methods have been widely used in medical and biological disciplines to analyse and filter complex databases [26-30]. They make it possible to synthesise data complexity and define clusters without using any *a priori *knowledge of the biological reasons for the existence of groups [27]. Furthermore, this statistical grouping took into account health conditions that could potentially affect several bodily systems and have various causes. Such conditions are common in wildlife [10]. In addition, we did not stratify the data analysis by species, so that disease processes potentially affecting several species (e.g. intoxications) could be recognised.

Below, we describe and discuss the application of a three-step statistical analysis and classification procedure for wildlife necropsy data, and the biological significance and value of the clusters obtained for syndrome definition.

## Methods

### Material

Wildlife disease surveillance in France has yielded over 53,000 records since 1986, through a nationwide network called SAGIR, managed by the French Hunting and Wildlife Agency (Office national de la chasse et de la faune sauvage, ONCFS), with input from national and departmental hunting federations (Fédération nationale des chasseurs, FNC, and Fédérations départementales des chasseurs, FDC) [22,31,32]. Cadavers of free-ranging wild terrestrial mammals and birds are reported to the network by hunters and the public. The people in charge of surveillance at departmental level select carcasses according to their state of preservation and relevance and bring them to the departmental veterinary diagnostic laboratory for *post-mortem *examination and, in some cases, for further biological analyses. Up to now, 252 different species and 228 causes of death have been diagnosed by 97 labs and registered in the national database.

From the data collected up to 31.12.2007, 23,228 cases had a registered description of macroscopic *post-mortem *findings (each case represented a wild animal cadaver reported to the network and submitted for laboratory examination). For the cluster construction process, we selected a subset of 8,709 cases, analysed between 1.1.1986 and 31.12.1997, for which a complete description of *post-mortem *findings was available. Unfortunately, the registrations in the database of *post-mortem *findings of some of the remaining 14,519 cases were incomplete because since 1998, lesions typical of certain causes of death have no longer been registered; the descriptions were later completed by data imputation and cases were then classified (see Discussion section).

Macroscopic lesions were described for each case, according to the affected organs (*Topography*) and their morphological characteristics (*Morphology*) indicating the changes observed in the organs. In addition, a *Cause of death *was registered for each case (including some for which no diagnosis was reached, labelled DNR). *Pathogenic agents *(bacteria, parasites, fungi, viruses, toxins), which were not necessarily related to the cause of death, were described for 75% of the cases. *Species *were recorded with their common name. In the original database, the terms used for *Cause of death*, *Morphology*, *Topography*, and *Pathogenic agent *were numerous and heterogeneous, so experts (see acknowledgements) and other sources of reference (College of Pathological Anatomy of Marseille, http://medidacte.timone.univ-mrs.fr/webcours/umvf/anapath/corpus.htm; Systematic Nomenclature of Medicine SNOMED CT, http://www.ihtsdo.org; Canadian Cooperative Wildlife Health Centre, http://wildlife1.usask.ca) were consulted to group them into broad categories. For the *Cause of death*, *Pathogenic agent *and *Species *classifications, all broad categories whose frequency was below 100 were combined into a single category named '*Other*'. The terms for *Topography *and *Morphology *were pooled according to their meaning, to obtain sufficient group sizes for statistical analysis (each term had to represent more than 3% of the total number of cases). *Topography *(15 modalities with two expressions each, either "not affected" or "affected") and *Morphology *(14 modalities with two expressions each, either "yes" or "no") were used to partition the data (active variables); their distributions are described in Tables 1 and 2. *Cause of death *(19 modalities), *Pathogenic agent *(18 modalities) and *Species *(9 modalities) were used for cluster interpretation (illustrative variables). The distributions of these variables are described in Tables 3, 4 and 5.

**Table 1:** Description of the variables for Topography

Modalities	Number affected	Proportion affected (%)
trachea	2,407	27.6
lung and bronchus	4,335	49.7
respiratory organs	1,200	13.8
stomachs	737	8.5
intestines	2,638	30.3
liver, pancreas	3,822	43.9
digestive organs	876	10.1
spleen	2,099	24.1
lymph nodes	910	10.5
urinary/genital organs	2,003	23.0
central nervous system, eye	513	5.9
skin and annexes	622	7.1
heart	738	8.5
musculoskeletal system	1,248	14.3
several organs	600	6.9

**Total**	**24,748**	

**Table 2:** Description of the variables for Morphology

Modalities	Number affected	Proportion affected (%)
abnormal colour or deposits	507	5.8
tumours, displacements, fibrosis	397	4.6
congestion	3,828	43.9
haemorrhage	2,948	33.8
haematoma	946	10.9
hypertrophy	2,299	26.4
other inflammation	2,587	29.7
abscesses and purulent inflammation	2,207	25.3
oedema, transudation	707	8.1
altered texture	1,505	17.3
necrosis, ulceration	818	9.4
parasitic lesions	595	6.8
signs of diarrhoea	753	8.7
traumatic lesions other than bleeding	802	9.2

**Total**	**20,899**	

**Table 3:** Distribution of the variable Cause of death

Modalities	Number affected	Proportion (%)
Diagnosis not reached (DNR)	2,354	27.0
Other trauma	961	11.0
Yersiniosis	774	8.9
VHD	551	5.9
EBHS	449	5.2
Pasteurellosis	436	5.0
Other internal parasitism	360	4.1
Respiratory infection, not specified	358	4.1
Shooting accident	323	3.7
Coccidiosis	251	2.9
Enterotoxaemia	219	2.5
Other septicaemia	210	2.4
Tularemia	209	2.4
Other poisoning	206	2.4
Anticoagulant poisoning	198	2.3
Other bacterial infection	176	2.0
Colibacillosis	136	1.6
Other	578	6.6

**Total**	**8,709**	

**Table 4:** Distribution of the variable Pathological agent

Modalities	Number affected	Proportion (%)
Strongylida	2,892	23.2
Eucoccidiorida	2,810	22.5
Escherichia	1,189	9.5
Trichuridae	967	7.7
Yersinia sp.	795	6.4
Pasteurellaceae	684	5.5
Clostridium	403	3.3
Ticks	384	3.1
Staphylococcaceae	332	2.7
Trematodes	249	2.0
Francisella	210	1.7
Trichomonas	163	1.3
Maggots	149	1.2
Corynebacteriaceae	149	1.2
Anticoagulants	133	1.1
Streptococcaceae	132	1.1
VHD-virus	100	0.8
Other	744	6.0

**Total**	**12,485**	

**Table 5:** Distribution of the variable Species

Modalities	Number affected	Proportion (%)
European brown hare	4,160	47.8
Roe deer	2,210	25.4
Wild rabbit	785	9.0
Wild boar	319	3.7
Wood pigeon	163	1.9
Alpine chamois	158	1.9
Red fox	157	1.9
Mallard	110	1.3
Other	1,072	12.2

**Total**	**8,709**	

### Method

*Topography *and *Morphology *(active variables) were used to perform a three step clustering in order to identify groups. First the data from each case were pre-processed by a multiple correspondence analysis (MCA) and reduced to their principal components. Then hierarchical ascendant clustering (HAC) was performed to determine a consistent partition. Finally the k-means algorithm was applied to consolidate this partition. The *Cause of death*, *Pathogenic agent *and *Species *variables were used to interpret the groups obtained (illustrative variables). Calculations were performed with R software (R Development Core Team (2009). R: A language and environment for statistical computing. R Foundation for Statistical Computing, Vienna, Austria. ISBN 3-900051-07-0, http://www.R-project.org). The packages, functions and references used for each process are indicated below.

#### Multiple correspondence analysis

MCA is a descriptive analysis of multidimensional qualitative data [33,34]. It allows the analysis of a matrix of I individuals depicted by J qualitative variables. Projections of these individuals in a J-dimensional space are used to calculate factorial axes, the first one retaining the maximum variance, and the following axes retaining the residual variance and being perpendicular to each other. MCA allows continuous quantitative coordinates to be attributed to individuals, and the most significant factorial axes to be selected, in order to reduce the number of dimensions of the initial space [35]. The variables' contributions to the axes are examined to visualise what they represent and to check for outliers. The number of axes to be retained is chosen, with respect to their meaning, so that the cumulated percentage of explained variance, calculated with the Benzécri method [36], is greater than 95%. MCA was performed with the R package "FactoMineR" [37].

#### Classification

**Figure 1:**
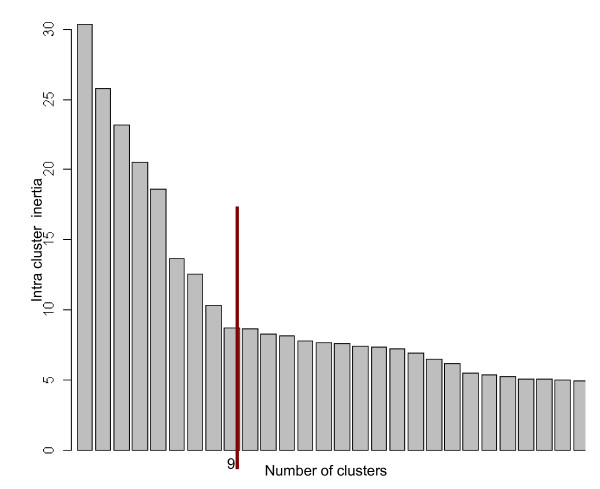
**Bar chart of the sum of intra-cluster inertias for different numbers of clusters**. The red line indicates the point of the changing slope.

HAC allows individuals to be grouped according to their coordinates, by calculating pair-wise distances between cases and aggregating the closest ones. We used the Euclidean distance [26], and the Ward criterion was used for aggregation, because it maximises inter-cluster variance while minimising intra-cluster variance [27,38]. Intra-cluster inertia is measured by the sum of the squares of the Euclidean distances between cluster cases and the cluster centroid. The closer the cases are grouped around the cluster centroid, the lower the intra-cluster inertia. The number of clusters to consider was determined classically by inspecting the bar chart of global intra-cluster inertia as a function of cluster numbers (Figure 1). The optimal number corresponds to the bar whose height-difference with respect to the preceding (*i.e*. to the left) bar in the chart is great compared to the height-difference with the following bars (*i.e*. to the right), indicating that a smaller number of clusters implies a sharp increase in intra-cluster variance. This choice was further supported by analysing the biological significance of the clusters at different levels of the clustering tree [27]. HAC was performed with the "agnes" function of the R package "cluster" [39].

As HAC clustering is not optimal due to the constraint of hierarchical grouping, the cases were then partitioned into the defined clusters by the k-means method [26], using the cluster centroids calculated by the HAC as seeds [38,40]. The k-means algorithm attributes cases to their nearest centroid. The cluster centroids are adjusted and calculations reiterated until no further significant improvement in intra-cluster inertia is achieved. K-means was performed with the "k-means" function of the R package "stats" [41].

The quality of the clustering result is highest when clusters are compact around their centroid and well separated from each other. This clustering quality is evaluated by a criterion defined as R^2 ^= 1-(sum of intra-cluster inertias/total inertia of the data set) [35]. The closer R^2 ^is to one, the better the clustering.

#### Cluster interpretation

The classification assigns each individual, *i.e*. an MCA-derived representation of a case, to a cluster.

**Figure 2:**
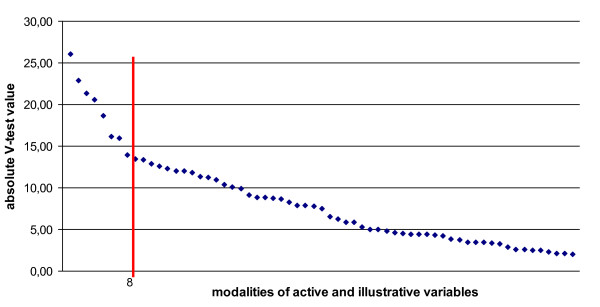
**Ordered absolute V-test values of significant modalities of active and illustrative variables of cluster 1**. The red line indicates the point of the changing slope.

In order to understand the meaning of these groups, one has to know which features characterise them. Cluster interpretation was based on both kinds of variables, the active and illustrative ones. The proportions of the modalities in each cluster and in the whole dataset were compared (V-test) [38]. We used a curve of ordered absolute V-test values for each cluster, and the point of the changing slope separated the more meaningful modalities from the other ones (Figure 2). Modalities with V-test values above the slope change were retained for cluster description [33]. Positive V-test values represent positive associations, negative values represent negative associations. Visualisation by projection of the modalities of the variables onto the factorial planes was also helpful for interpretation.

## Results

We used the 14 modalities of *Morphology *and 15 modalities of *Topography *from our dataset of 8,709 cases (*i.e*. recorded mortality cases from 1986 to 1997 with a description of *post-mortem *findings) to build a statistical classification (active variables). The first five axes of the MCA loaded more than 96% of the total variance of the 29-dimensional space. Details are given in Table 6. Variables contributing to axis definition differed from one axis to the other, and no rare modalities had a determining influence.

**Table 6:** Variances of the first eleven factorial axes

Eigenvalue (B) ^a^	Variance (B) ^b ^(%)	Cumulated variance (B) ^c ^(%)
4.91 e-03	62.41	62.41
1.15 e-03	14.62	77.03
6.67 e-04	8.45	85.48
4.53 e-04	5.74	91.22
3.95 e-04	5.01	96.23
1.77 e-04	2.24	98.47
7.80 e-05	0.99	99.46
2.51 e-05	0.32	99.78
1.38 e-05	0.17	99.95
2.41 e-06	0.03	99.98
1.40 e-06	0.02	100.00

**^a ^**Eigenvalue with Benzécri correction

**^b ^**Percentage of variance with Benzécri correction

**^c ^**Cumulated % of variance with Benzécri correction

HAC was then performed on the case-coordinates derived from the first five axes of the MCA. Analysis of intra-cluster inertia levels of the clustering-tree (Figure 1), and the examination of the biological significance of clusters at different thresholds, indicated that nine was the optimal number of clusters. With a higher number of clusters, cases which were very similar from a biological point of view would have been separated, while fewer clusters would have merged cases exhibiting rather different lesional features.

Partition strengthening by the k-means method (10 iterations) was used to attribute the cases to these nine clusters. The calculated R^2 ^value was 0.62.

The modalities of variables (active and illustrative) best describing the different clusters are presented in Table 7. To analyse to what extent a cluster could be considered as a syndrome in terms of pathological findings, our interpretation was based on these descriptions, and on pathological descriptions and differential diagnoses found in the literature for the more frequent *Causes of death *of the clusters.

**Table 7:** Cluster description by active and illustrative variables

Cluster	Size	Type of variable	Modality	% cluster^a^	% global^b^	V test
**1**	1057	active	haemorrhage = yes	70.6	33.9	26.1
			hypertrophy = no	98.0	73.6	22.9
			other inflammation = no	95.0	70.3	21.3
			spleen = not affected	97.4	75.9	20.5
		
		illustrative	cause of death = anticoag. pois.	12.8	2.3	18.7
			agent = anticoagulants	9.1	1.5	16.2
			species = roe deer	9.3	25.4	-14.0

**2**	1117	active	diarrhoea = yes	46.5	8.6	infinite
			intestines = affected	80.9	30.3	37.8
		
		illustrative	cause of death = coccidiosis	11.3	2.9	14.6
			cause of death = other int. paras.	10.4	4.1	9.8
			cause of death = other poisoning	6.0	2.4	7.4
			cause of death = DNR	36.3	27.0	7.3

**3**	1632	active	haemorrhage = no	99.3	66.1	37.5
			spleen = not affected	99.0	75.9	29.2
			trachea = not affected	97.2	72.4	29.0
			liver = not affected	86.3	56.1	28.7
			congestion = no	86.0	56.0	28.5
			hypertrophy = no	95.9	73.6	25.9
			altered texture = no	99.0	82.7	23.4
		
		illustrative	cause of death = other	16.2	6.6	15.4
			species = wood pigeon	5.6	1.9	10.6
			cause of death = resp. infection	8.7	4.1	10.6
			cause of death = shoot. accident	9.3	4.1	10.5
			species = roe deer	32.9	25.4	7.6
			cause of death = VHD	0.4	3.6	-9.3
			species = European brown hare	31.1	47.8	-15.1
			cause of death = EBHS	0.3	7.5	-15.5

**4**	518	active	stomach = affected	51.0	8.5	26.7
			other inflammation = yes	75.7	29.7	22.3
			heart = affected	41.7	8.5	21.6
			parasitic lesions = yes	34.9	6.8	19.8
			transudates = yes	32.8	8.1	16.9
			necrosis = yes	33.0	9.4	15.5
			lung = affected	81.7	50.2	15.3
		
		illustrative	agent = strongylida	75.7	33.2	20.4
			species = roe deer	64.1	25.4	19.2
			agent = trichurida	32.2	11.1	13.4
			species = European brown hare	10.8	47.8	-18.5

**5**	750	active	other inflammation = yes	77.2	29.7	28.2
			lung = affected	92.1	50.2	25.8
			heart = affected	29.7	8.5	18.0
		
		illustrative	agent = pasteurellaceae	20.1	7.9	11.3
			cause of death = resp. infection	13.2	4.1	10.8
			cause of death = pasteurellosis	14.0	5.0	10.0
			species = wild boar	8.4	3.7	6.3

**6**	892	active	traumatic lesions = yes	75.2	9.2	infinite
			musculoskeletal system = yes	87.6	14.3	infinite
			haematoma = yes	41.7	10.9	25.9
		
		illustrative	cause of death = other trauma	49.7	11.0	31.6
			species = roe deer	48.3	25.4	15.6
			cause of death = shoot. accident	9.5	3.7	8.3

**7**	808	active	trachea = affected	84.9	27.6	36.0
			haemorrhage = yes	84.3	33.9	31.2
			altered texture = yes	51.9	17.3	23.9
			liver = affected	80.0	43.9	22.0
			congestion = yes	78.6	44.0	21.1
			lung = affected	83.8	50.2	20.8
			respiratory organs = affected	38.5	13.8	18.6
		
		illustrative	cause of death = EBHS	30.7	7.5	21.2
			cause of death = VHD	19.4	3.6	19.2
			species = wild rabbit	25.4	9.0	14.6
			cause of death = other trauma	2.7	11.0	-9.1
			species = roe deer	6.4	25.4	-14.6

**8**	1306	active	spleen = affected	87.9	24.1	infinite
			hypertrophy = yes	89.4	26.4	infinite
			liver = affected	66.3	43.9	17.7
			purulent inflammation = yes	44.7	25.3	16.6
		
		illustrative	cause of death = yersiniosis	34.2	8.9	29.3
			species = European brown hare	82.4	47.8	28.0
			agent = yersinia	32.8	9.1	27.3
			cause of death = other trauma	4.1	11.0	-9.6
			species = roe deer	6.8	25.4	-18.6

**9**	629	active	hypertrophy = yes	84.7	26.4	32.1
			spleen = affected	81.6	24.1	31.8
			trachea = affected	77.6	27.6	27.2
			altered texture = yes	63.1	17.3	27.0
			urinary/genital organs = affected	70.9	23.0	26.7
			congestion = yes	89.8	44.0	25.1
			liver = affected	88.6	43.9	24.3
			lymph nodes = affected	45.5	10.4	23.9
			haemorrhage = yes	71.5	33.9	20.0
		
		illustrative	species = European brown hare	72.3	47.8	12.9
			cause of death = EBHS	23.1	7.5	12.9
			agent = eucoccidiorida	52.5	32.3	10.8
			cause of death = other trauma	2.7	11.0	-8.0
			species = roe deer	12.7	25.4	-8.0

**^a ^**Frequency of modality in cluster

**^b ^**Frequency of modality in the whole dataset

Higher V-test values, p < 0.05

Cluster 1 comprised 12.1% of the total number of cases. It was characterised by haemorrhagic lesions, associated with evidence of anticoagulant compounds. Haemorrhage is also present for example in trauma cases or in the European brown hare syndrome (EBHS), but in Cluster 1 all other types of lesions were absent. For anticoagulant poisoning, evidence of massive bleeding is noted at necropsy and the lack of coagulation is highly suggestive of exposure [42]. According to Berny [43], large herbivores are usually less susceptible than predators, which was highlighted here by a negative association of this cluster with roe deer (*Capreolus capreolus*).

Animals in Cluster 2 (12.8% of cases) presented diarrhoea and lesions of the gut, sometimes with parasitism, namely coccidiosis, but some were intoxicated by toxic agents. These agents were mostly cholinesterase inhibitors, which is consistent with symptoms of diarrhoea [42].

Cluster 3 was the largest cluster (18.7% of cases). It grouped cases characterised by the absence of lesions typical of Clusters 7 and 9, and was associated with rarer causes of death, such as those grouped under "Other", respiratory infections of wood pigeons (*Columba palumbus*) or roe deer shooting accidents. It is therefore difficult to propose a straightforward biological explanation for this cluster.

Cluster 4 (5.9% of cases) was typed by different locations and types of parasitism to Cluster 2. Inflammatory, necrotic or parasitic lesions of the stomach, lung and heart associated with the presence of *Strongylida *(in 76% of cases) or *Trichurida *were found in this cluster, mainly observed in roe deer. Debilitating conditions, such as heavy parasite burden, especially in the stomach can cause mucosal abrasions that promote the action of toxigenic bacteria, leading to enterotoxaemia or septicaemia [44].

Cluster 5 (8.6% of cases) identified inflammatory bacterial diseases of thoracic organs, in particular pasteurellosis. The health conditions in this cluster affected 19.8% of the wild boar (*Sus scrofa*), in the analysed dataset. Typical findings included pleuro-pneumonia, purulent bronchitis, fibrinous pleurisy and pericarditis [44,45].

Cluster 6 (10.2% of cases) dealt with traumatic lesions, especially in roe deer.

Cluster 7 (9.3% of cases) was defined by an altered texture and haemorrhagic and congestive lesions of the trachea, liver and lungs and was linked to Viral hemorrhagic disease (VHD) and EBHS as causes of death, and to rabbits (*Oryctolagus cuniculus*) (25% of cases in this cluster) and hares (*Lepus europaeus*) (64%). Caliciviruses that cause EBHS and VHD are closely related and both induce similar pathological changes [46].

Cluster 8 (15.0% of cases) was characterised by hypertrophy and purulent lesions of the spleen and liver. In this cluster they appeared linked to hares and to yersiniosis and *Yersinia pseudotuberculosis*, and to a lesser degree to tularemia. Acute yersiniosis is characterised by an enlarged spleen and necrotic hypertrophied mesenteric lymph nodes; the chronic form causes multiple small nodular caseous lesions of the spleen, liver, and possibly kidneys, lungs and cecum [44,47,48]. Similar lesions of the spleen and liver can be found in tularemia and yersiniosis, which might explain why these two diseases were grouped together.

Cluster 9 (7.2% of cases) and Cluster 7 had rather similar characteristics. Hypertrophy of the spleen and lesions of the kidney and lymph nodes were present in Cluster 9 but not in Cluster 7. Cluster 9 was also linked to EBHS and hares, as well as to tularemia and *Eucoccidiorida*. Liver coccidiosis, tularemia and haemorrhagic septicaemia (due to *Pasteurella *sp.) are differential diagnoses to EBHS [49]. As hares and EBHS were associated with both clusters, they possibly reflect two different stages of the same disease (acute or protracted) in this host [50].

## Discussion

This paper describes the use of a three-step clustering method to group cases of wild animals found dead with similar *post-mortem *findings, over a period of ten years in France, for syndrome definition.

The SAGIR network continuously collects data from investigations of causes of mortality in free-ranging animals in France. However, there is some variability in the intensity of surveillance both spatially and among species, which influences the representativeness of the database. The network provides a more accurate picture of health events for game species than for non-game animals [31]. Furthermore, the network's activity is uneven from one *département *to another. Nevertheless, these differences have been relatively stable over time, so the quantity and quality of data appeared suitable for trend analysis and detection of unusual health events [51].

Despite the fact that laboratory staff involved in the network has been regularly trained in *post-mortem *examination of wildlife cadavers, differences in the precision of descriptions contributed to the complexity of the database. Nevertheless, these descriptions were assumed to be more reliable than diagnostic conclusions, because the process of arriving at a cause of death did not follow a standardised procedure.

Methods of classifying qualitative variables are dependent on the number of occurrences for each modality, and small counts make a minor contribution to the variance of the factorial axes [38]. The number of terms used for coding the variables was reduced by preliminary work, and we tried to minimise the risk of misinterpretation by relying on the skill of experts and other sources of reference. For statistical reasons some categories had to be combined further (e.g. "genital organs" alone were mentioned only 193 times, so they were combined with "urinary organs"). For some other categories, the descriptions were more or less detailed (e.g. "respiratory organs" instead of "lung" or "trachea"). We decided not to group these categories together, in order to keep as much precision as possible. These choices may have influenced the outcomes of the classification. However, results were consistent, as "respiratory organs" together with "lung" and "trachea" were determining for Cluster 7, "lung" alone was determining for Clusters 4 and 5, and "trachea" alone for Cluster 9.

Variables were split into active and illustrative ones to avoid redundancy and limit insignificant noise, produced for example by information that was not necessarily linked to the case's cause of death. Noise reduction was also the reason for retaining only the coordinates on the first five axes of the MCA. These axes were used regardless of their rank, because each represented very different biological information that retained the most differentiating characteristics of the dataset.

The statistical classification procedure used here showed its ability to handle large datasets and identify pathologically relevant characteristics. However, it should be noted that the cluster description does not address the full range of lesions found on an animal. It merely indicates features that are characteristic and allow clusters to be distinguished. As a result, the cases which were infrequent or poorly defined were gathered in a cluster (Cluster 3) that is difficult to qualify as an entity. Diseases that remained rare or those that induced only unspecific lesions, such as congestion of different organs, could not be highlighted by our approach.

The clusters obtained in this study were of three different types: those which were species- and disease-specific (Clusters 7, 8 and 9), those suggestive of specific conditions but not species-specific (Clusters 1, 5 and 6), and the others, covering a broad pathologic condition (Clusters 2 and 4). It might be interesting to group Clusters 7 and 9 for further epidemiological analysis as they seem to present two different views of the same disease.

The characteristics of the clusters derived from our analysis are consistent with features found in previous epidemiological studies on wildlife diseases in this country [42,52-55]. The clusters reflect the most distinct and most frequent disease entities on which the surveillance network focused. The importance of investigations into VHD and EBHS for example, which were emerging diseases in the early 1990 s [50,56], was decisive in defining two clusters.

The statistical classification of cases collected by the French SAGIR network could lead to the adoption by the surveillance community of eight distinct syndromes:

1) a hemorrhagic syndrome, interesting because it allows accidental wildlife intoxications to be monitored [42] and could potentially also detect anthrax cases [16];

2) an enteritic/diarrheic syndrome, which could reflect environmental constraints, such as changes in food supply [57] or density related parasite burdens [58,59];

3) a multifactorial (parasites and toxigenic bacteria) syndrome, more specific to the difficult living conditions of wild ruminants [55];

4) a respiratory syndrome, which is a disease complex that takes a regular toll on wildlife [44];

5) a trauma-related syndrome, representing one of the foremost causes of death in our database, but less interesting from an epidemiological point of view;

6) a syndrome of acute hepatitis-like diseases, which reflects the importance of EBHS and VHD, especially during the study period, and could be useful for other emerging hepatites;

7) a syndrome of subacute or chronic diseases of the liver, kidney and spleen, caused mostly by endemic bacteria. This syndrome could be useful for the monitoring of tularemia and salmonella outbreaks, potentially threatening public health [60,61];

8) a miscellaneous syndrome; despite being difficult to understand, this syndrome is worth considering, because an unknown disease might probably first increase this group before being recognised as a distinct entity.

Future cases can be attributed to the defined syndromes by determining their MCA-derived representation and the cluster they belong to [40]. We used this procedure on the remaining 14,519 cases collected between 1998 and 2007. Missing information was completed statistically by multivariate imputation. MCA with the above determined eigenvalues was used to calculate the coordinates of these additional cases in the five-dimensional space. These coordinates were used to determine the cluster to which each case belonged (smallest Euclidean distance to cluster centroid). Clustering quality of the whole dataset (R^2 ^= 0.605) was not substantially different from that of the initial dataset (R^2 ^= 0.62) (unpublished work).

As new diseases with distinct pathological profiles emerge in free-ranging wild animals over time, the syndrome definition might evolve. The statistical classification could be revised in the future, and historical data could be integrated in the classification process, thus allowing the analysis of continuous time series.

For the epidemiological study of the syndromic time series, we will develop models and anomaly detection algorithms on the number of cases of each syndrome per time unit from the historical database [62].

## Conclusions

The results presented above suggest that macroscopic necropsy findings are valuable for identifying distinct pathological profiles among wild animal carcasses collected by a general surveillance network. The construction of our typology was based on an unsupervised statistical approach; it allowed an impartial reduction of all the information present in our complex dataset and then a robust classification. This approach identified meaningful clusters, reflecting the most frequent disease groups in the database and their distinctive characteristics. To our knowledge this is the first time that this method has been used to construct clusters from animal necropsy data.

Cluster characteristics lead to the definition of eight syndromes that could classify all the investigated cases and potentially reflect all disease events including new diseases. Moreover, some of these syndromes referred to pathological entities that go beyond species and specific diseases, and could reflect environmental stresses on wildlife. Others could be used for the surveillance of zoonoses. Cluster and subsequently syndrome definition were however dependent on the focus of the surveillance network which provided the data we used.

Syndromic classification of cases based on their pathological profile has practical value because it does not need a precise diagnosis and therefore provides a rapid, reliable and rather inexpensive means of analysing wildlife health data.

This approach could improve the usefulness and cost-effectiveness of existing wildlife mortality monitoring systems.

## Authors' contributions

EWP conceived the study, carried out the data description, statistical classification and drafted the manuscript. EM participated in the design of the study and wrote the scripts for the statistical analyses. MA and DC participated in study design and coordination and helped to draft the manuscript. All authors read and approved the final manuscript.
